# Geriatric fragility fractures are associated with a human skeletal stem cell defect

**DOI:** 10.1111/acel.13164

**Published:** 2020-06-14

**Authors:** Thomas H. Ambrosi, L. Henry Goodnough, Holly M. Steininger, Malachia Y. Hoover, Emiley Kim, Lauren S. Koepke, Owen Marecic, Liming Zhao, Jun Seita, Julius A. Bishop, Michael J. Gardner, Charles K. F. Chan

**Affiliations:** ^1^ Department of Surgery Stanford Medicine Stanford CA USA; ^2^ Institute for Stem Cell Biology and Regenerative Medicine Stanford Medicine Stanford CA USA; ^3^ Department of Orthopaedic Surgery Stanford Hospital and Clinics Stanford CA USA; ^4^ Medical Sciences Innovation Hub Program RIKEN Tokyo Japan

**Keywords:** aging, bone healing, geriatric fractures, human skeletal stem cell, sexual dimorphism

## Abstract

Fragility fractures have a limited capacity to regenerate, and impaired fracture healing is a leading cause of morbidity in the elderly. The recent identification of a highly purified bona fide human skeletal stem cell (hSSC) and its committed downstream progenitor cell populations provides an opportunity for understanding the mechanism of age‐related compromised fracture healing from the stem cell perspective. In this study, we tested whether hSSCs isolated from geriatric fractures demonstrate intrinsic functional defects that drive impaired healing. Using flow cytometry, we analyzed and isolated hSSCs from callus tissue of five different skeletal sites (*n* = 61) of patients ranging from 13 to 94 years of age for functional and molecular studies. We observed that fracture‐activated amplification of hSSC populations was comparable at all ages. However, functional analysis of isolated stem cells revealed that advanced age significantly correlated with reduced osteochondrogenic potential but was not associated with decreased in vitro clonogenicity. hSSCs derived from women displayed an exacerbated functional decline with age relative to those of aged men. Transcriptomic comparisons revealed downregulation of skeletogenic pathways such as WNT and upregulation of senescence‐related pathways in young versus older hSSCs. Strikingly, loss of Sirtuin1 expression played a major role in hSSC dysfunction but re‐activation by trans‐resveratrol or a small molecule compound restored in vitro differentiation potential. These are the first findings that characterize age‐related defects in purified hSSCs from geriatric fractures. Our results provide a foundation for future investigations into the mechanism and reversibility of skeletal stem cell aging in humans.

## Graphical abstract

Human skeletal stem cells (hSSCs) are present and accumulate at fracture sites independent of age. Impaired healing in the elderly might underlie functional defects in hSSCs which are exacerbated in women. Re‐activation of Sirt1 rejuvenates geriatric hSSCs independent of sex.

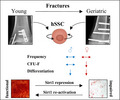

## 1 INTRODUCTION

Geriatric fragility fractures are exceedingly common, with an estimated 6.3 million projected to occur annually by the year 2050 (Friedman & Mendelson, 2014). In 2010 alone, geriatric fracture care cost an estimated $17 billion in the United States (Becker, Kilgore, & Morrisey, 2010). Geriatric fractures have increased complication rates after surgical treatment, with significant rates of implant‐related complications, re‐operation, and mortality (Carpintero et al., 2014; Schnell, Friedman, Mendelson, Bingham, & Kates, 2010). Given the increasing incidence, significant financial burden, and adverse effects on patients, new approaches are urgently needed to improve the treatment of geriatric fractures.

Bone is a unique tissue in terms of its normally excellent regenerative capacity after fracture injuries. Extensive genetic and biological studies in mice have detailed the major stages of fracture healing which progress from inflammation to hematoma then soft and hard callus, and subsequent remodeling (Schell et al., 2017). Additionally, decades of clinical experience have described many of the pathologies affecting fracture healing from both diagnostic and therapeutic perspectives (Mirhadi, Ashwood, & Karagkevrekis, 2013). Bringing together these perspectives and connecting them to the biology of stem cells in bone could deepen mechanistic understanding of the fracture healing process in humans that may translate to clinical advancements to improve fracture healing, particularly in the elderly.

The origins of the skeletal lineages and their relationship to a “mesenchymal stem cell” (“MSC”) remain poorly understood. Previous efforts to isolate a “MSC” have relied on the method of dissociated adhering cells to plastic cultureware in vitro to enrich for cells with fibroblast colony‐forming unit (CFU‐F) potential, apparent multi‐mesenchymal lineage differentiation (bone, fat, cartilage, muscle, and endothelial), and the ability to support hematopoiesis (Dominici et al., 2006). However, this technique is limited by its retrospective nature, generates cultures of multiple overlapping cell lineages, and ultimately yields heterogeneous populations of stem/progenitor and more mature cells, rather than a purified skeletal stem cell (SSC). The identification of cell surface markers subsequently refined the ability to enrich for true lineage‐restricted stem cells of distinct developmental origins including pericytes, stromal cells, muscle stem cells, as well as less proliferative lineage‐committed bone, cartilage, and hematopoietic stromal progenitors (Lv, Tuan, Cheung, & Leung, 2014; Pinho et al., 2013; Sacchetti et al., 2007; Sorrentino et al., 2008). Recently, we have prospectively isolated a bona fide human skeletal stem cell (hSSC) from fetal and adult human bones, which self‐renews in vitro and in vivo, and demonstrates multi‐lineage differentiation including bone, and cartilage cell fates, but not fat (Chan et al., 2018).

The involvement of osteogenic cells in human bone fracture repair has previously been demonstrated, although these early studies involved predominantly heterogeneous progenitor cell populations (Iwakura et al., 2009). Our group has also shown in mice that skeletal stem and progenitor cells are enriched in fracture calluses and that they are responsive to acute injury (Marecic et al., 2015; Tevlin et al., 2017). The effect of aging on the human SSC response to injury is unknown, although it has been proposed that aging results in intrinsic cellular abnormalities in resting/uninjured “MSCs” (Zhou et al., 2008) and there are conflicting results on age‐related changes in bone marrow stromal cell frequencies (Ganguly et al., 2019; Josephson et al., 2019; Maryanovich et al., 2018). At the molecular level, several studies have compared global gene expression and cytokine changes in bulk human fracture tissues in normal versus aging or osteoporotic fractures (Caetano‐Lopes et al., 2011; Kolar, Gaber, Perka, Duda, & Buttgereit, 2011), but it is not clear from these studies what the effects of aging on specific SSCs are. Finally, there is strong evidence for sexual dimorphism in skeletal regenerative abilities between men and women and older women experience a disproportionately high fracture risk compared to older men (Alswat, 2017; Parker, Raghavan, & Gurusamy, 2007; van Staa, Dennison, Leufkens, & Cooper, 2001). Whether age‐related changes in circulating hormonal levels between men and women affect hSSC function is also unclear though it has been reported for other stem cells (O’Brien, Soliman, Li, & Bilder, 2011; Velardi et al., 2018).

Here, we investigate whether geriatric fractures are associated with functional defects in hSSCs. Comparing normal versus geriatric hSSCs could identify molecular and functional differences that might serve as a basis for new therapeutic strategies aimed at improving the healing of geriatric fractures.

## 2 RESULTS

### 2.1 Human SSCs are present at multiple skeletal fracture sites and are amplified upon injury

**Figure 1:**
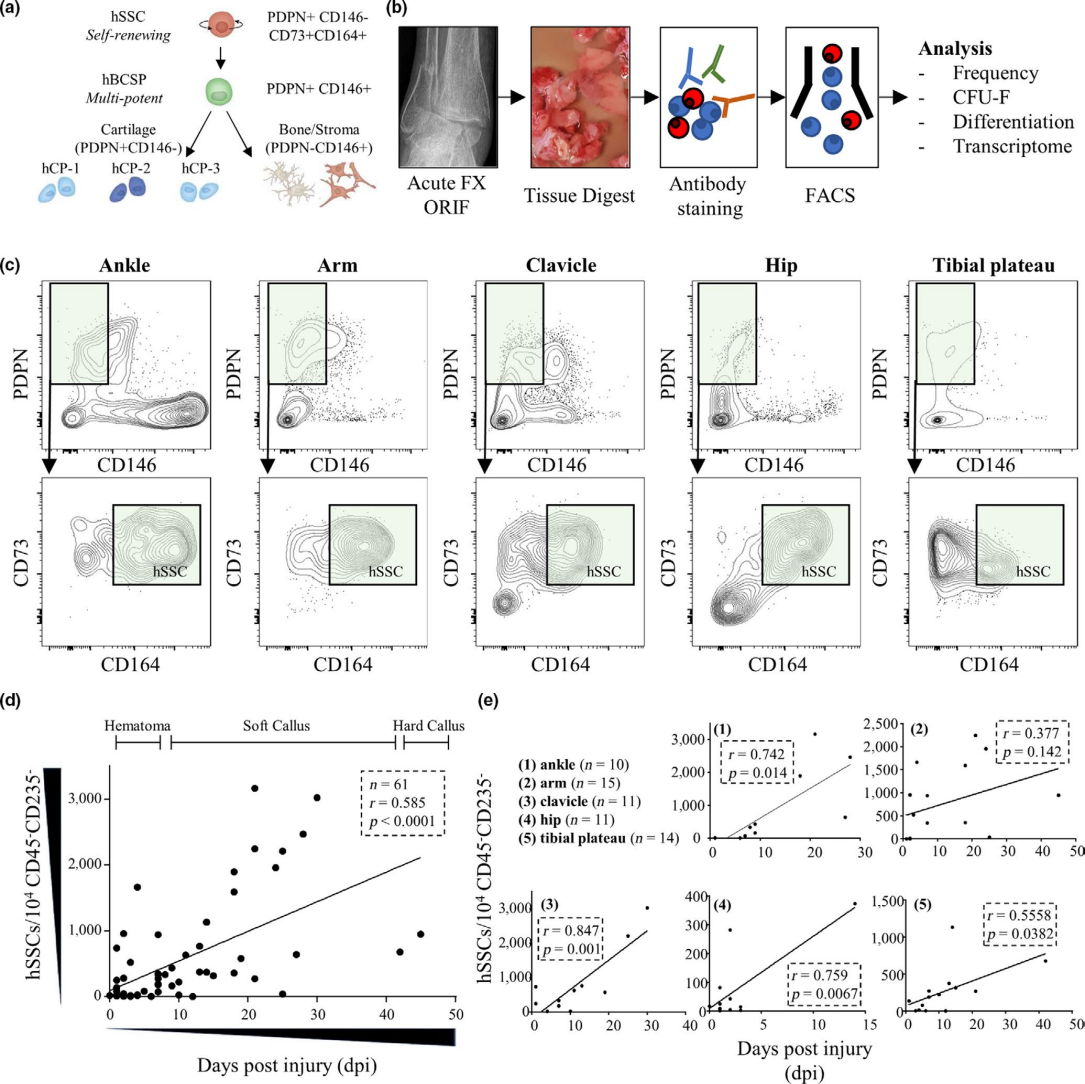
Human SSCs accumulate upon fracture injury. (a) The human skeletal lineage tree. Human skeletal stem cells (hSSCs) sit at the apex of the lineage tree self‐renewing and giving rise to downstream cell populations including human bone, cartilage, stroma progenitors (hBCSP), human chondrogenic progenitors (hCP‐1, hCP‐2, and hCP‐3), bone, and stroma cell types. All cell populations are negative for CD45, CD235a, CD31, and Tie2. PDPN: podoplanin. (b) Experimental overview for the present study. (c) Representative FACS plots gated from singlet living cells gated negative for CD45, CD235a, CD31, and Tie2 showing the presence of hSSCs in callus tissue of fracture sites from various skeletal regions. Cells gated for PDPN^+^ and CD146^−^ phenotype (upper panel) were further gated for CD73^+^ and CD164^+^ hSSCs (lower panel). (d) The correlation of hSSC prevalence and time (days post injury: dpi) is plotted for specimen used in this study. Demarcated healing stages are reflective of gross appearance of tissue at time of collection. (e) The same data are grouped and plotted by their origin of skeletal site. All graphs and data show two‐tailed Pearson correlation test results.

We have recently reported the identification of a highly purified hSSC giving rise to defined downstream lineages present in long bones (Figure 1a) (Chan et al., 2018). We now determined whether SSCs are involved in human fracture healing by testing for the presence of hSSCs at multiple distinct anatomical sites. We analyzed fracture callus tissue from 61 patients of all age groups (13–94 years) and from five of the most affected skeletal injury sites using flow cytometry and in vitro assays (Figure 1b, Table 1). Fluorescence‐activated cell sorting (FACS) analysis revealed that the presence of hSSCs was not limited to the proximal femur (hip) and they were also detected at other fracture sites (ankle, tibial plateau, arm, and clavicle) (Figure 1c). When we plated freshly sorted fracture hSSCs from all investigated skeletal sites, they displayed similar size and morphological characteristics (Figure S1a), implying that our marker profile enriches for hSSCs irrespective of the skeletal region. Next, we tested whether hSSCs increase in frequency at the fracture site during the healing process. We observed that hSSC frequency increased significantly in the initial days following injury during the onset of fracture healing (Figure 1d), albeit with exceptions depending on the specific fracture site (Figure 1e). The rate of hSSCs frequency increase corresponded with observed rates of fracture callus development in human fractures consistent with the involvement of hSSC in fracture repair (Einhorn, 2005).

**Table 1:** Summary of source tissues and respective demographics of patient specimens

Skeletal site (*n*)	Male	Female	Age (average)	Days post injury (average)
Ankle (10)	6	4	13–90 years (54)	1–28 (13)
Arm (15)	5	10	21–77 years (52)	2–45 (12)
Clavicle (11)	9	2	24–65 years (45)	1–30 (12)
Acetabulum (11)	2	9	26–94 years (69)	0–14 (3)
Tibial Plateau (14)	8	6	21–82 years (47)	1–42 (11)
Total (61)	30	31	13–94 years (53)	0–45 (10)

### 2.2 Geriatric hSSCs are functionally impaired at the differentiation level

**Figure 2:**
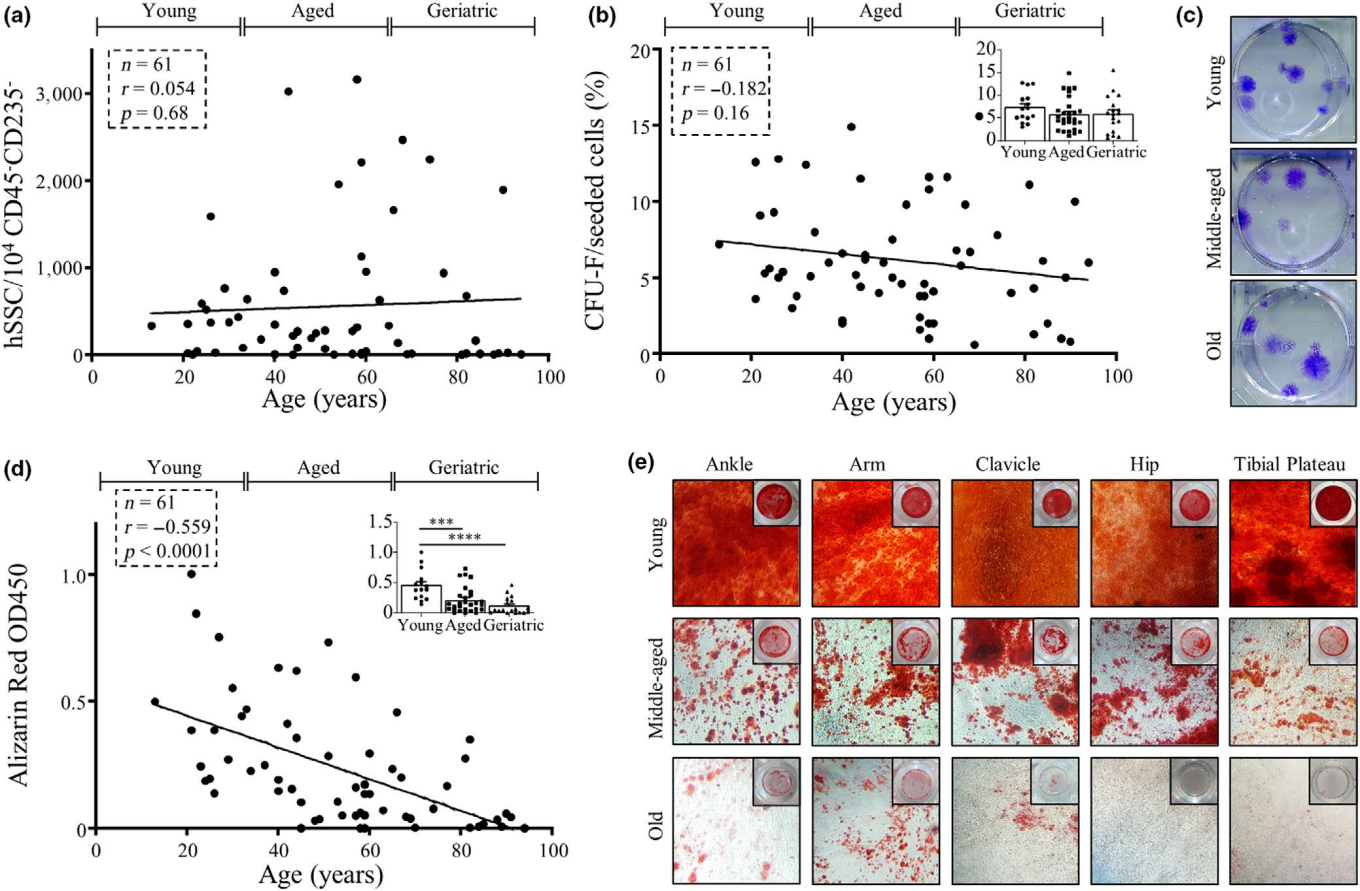
Geriatric fracture‐derived hSSC dysfunction occurs at the differentiation level. (a) The hSSC prevalence over donor age is plotted showing no correlation. (b) Fibroblast colony‐forming unit (CFU‐F) percentage is plotted over donor age. Top right, small plot: CFU‐F percentage was grouped by age into young (<35 years, *n* = 15), aged (35–65 years, *n* = 28), and geriatric (>65 years, *n* = 18). (c) Representative Crystal Violet staining for colonies formed by hSSCs derived from young, aged, and geriatric donors displaying similar quantities and sizes of colonies formed independent of age. (d) The correlation of osteogenic potential assessed by Alizarin Red S staining with donor age of fracture hSSCs is shown. Top right, small plot: Alizarin Red S staining measurement was grouped by age into young (<35 years, *n* = 15), aged (35–65 years, *n* = 28), and geriatric (>65 years, *n* = 18). (e) Representative Alizarin Red S stained culture images of differentiation outcome from young, aged, and geriatric donor fracture hSSCs from different skeletal sites are displayed. Linear correlations as analyzed with two‐tailed Pearson test. Grouped data plots are shown as mean + standard error of mean (*SEM*). Significance assessed by one‐way ANOVA (***p < .001 and ****p < .0001).

Since aging and osteoporosis are known drivers of gradual bone loss and poor regeneration in humans (Demontiero, Vidal, & Duque, 2012) (Figure S1b), we next tested for differences in hSSCs derived from aged/geriatric fractures relative to those isolated from fractures of younger individuals (<35 years). Surprisingly, neither the frequency nor the CFU‐F capacity changed significantly with increasing donor age (Figure 2a,b). Cell and colony sizes also were similar between skeletal and age groups, respectively (Figure 2c, Figure S1a). Furthermore, we did not observe a correlation between hSSC frequency and CFU‐F numbers (Figure S1c).

We then tested the osteochondrogenic differentiation potential of isolated hSSCs in vitro and found that expanded cells derived from older fracture‐derived hSSCs showed a significant loss in the capacity to mineralize as measured by Alizarin Red S staining (Figure 2d,e, Figure S1d). Importantly, we also observed a similar deficiency in chondrogenic capacity as measured by proteoglycan production in chondrogenic conditions by Alcian Blue staining (Figure S1e). The time from injury to hSSC isolation did not affect differentiation outcome (Figure S1f). Altogether, these findings indicate that the aging human skeleton retains hSSCs which accumulate in response to injury but have diminished bone‐ and cartilage‐forming potential.

### 2.3 Sex‐specific differences exacerbate the hSSC aging phenotype

**Figure 3:**
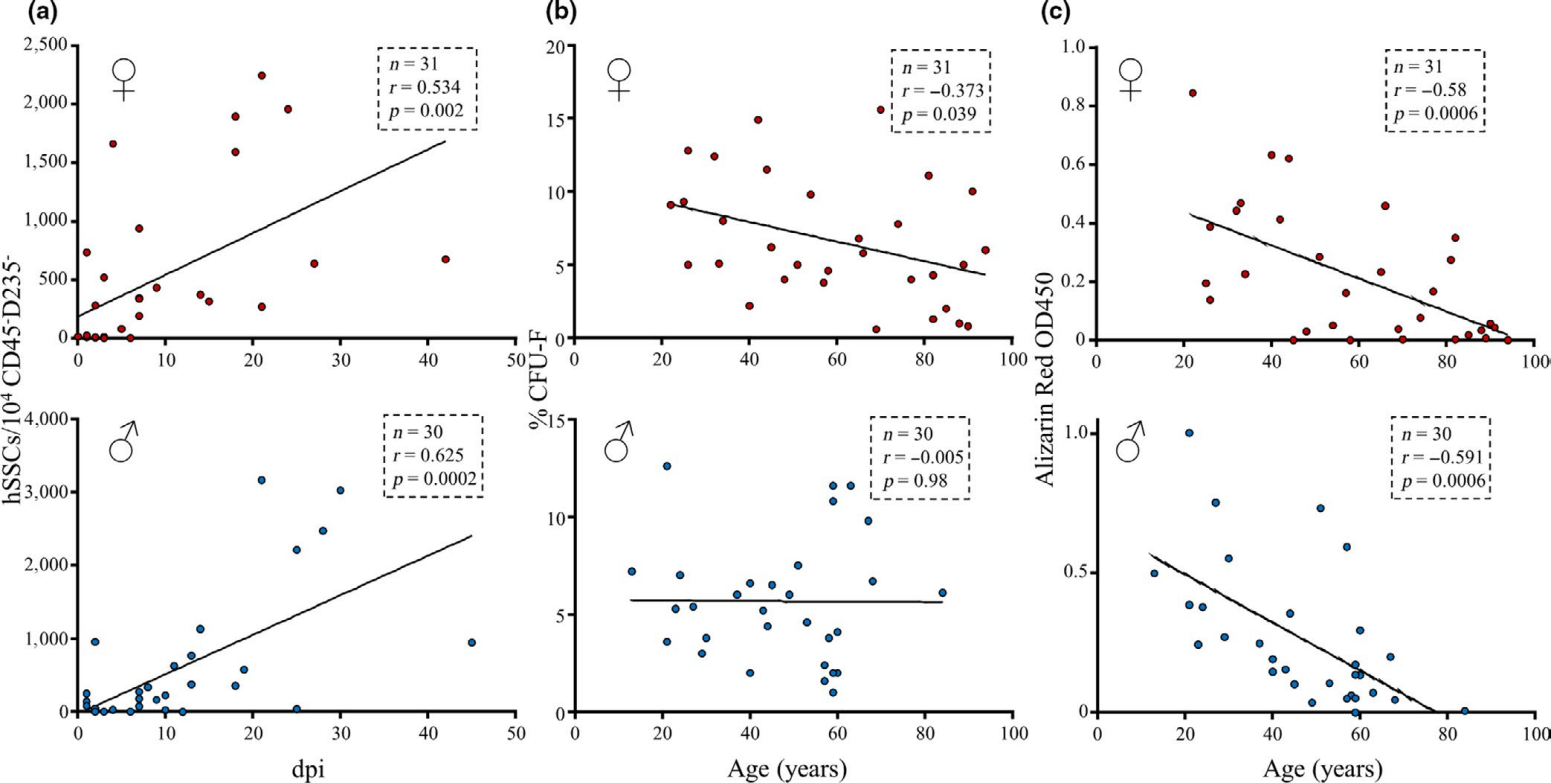
Female donor‐derived hSSCs display an exacerbated functional defect. (a) Correlation of hSSC prevalence at fracture site and days post injury (dpi) is shown (female: upper plot, male: lower plot). (b) Correlation of hSSC CFU‐F ability and donor age is shown (female: upper plot, male: lower plot). (c) Correlation of hSSC in vitro osteogenic potential and donor age is shown (female: upper plot, male: lower plot). Linear correlations as analyzed with two‐tailed Pearson test.

Since post‐menopausal women generally recover less well from fragility fractures than men (Cummings & Melton, 2002; Kempen, Sanderman, Scaf‐Klomp, & Ormel, 2003), we also investigated whether hSSCs from acute fractures of females demonstrate distinct phenotypes compared to male hSSCs during aging. We noted that injury‐associated expansion of hSSCs at the fracture site was comparable in both sexes (Figure 3a, Figure S2a). However, while advanced age in male‐derived hSSCs did not alter CFU‐F capability, female hSSCs displayed a significant decline (Figure 3b, Figure S2b). Mineralization ability as assessed by Alizarin Red S staining was impaired in hSSCs from women and men alike (Figure 3c, Figure S2c). Altogether, these observations suggest that age‐related defects are more pronounced in female hSSCs, which may underlie the greater probability of adverse fracture healing outcome in women.

### 2.4 Sirtuin1 re‐activation can rescue age‐associated impairment of hSSCs independent of sex and skeletal site

**Figure 4:**
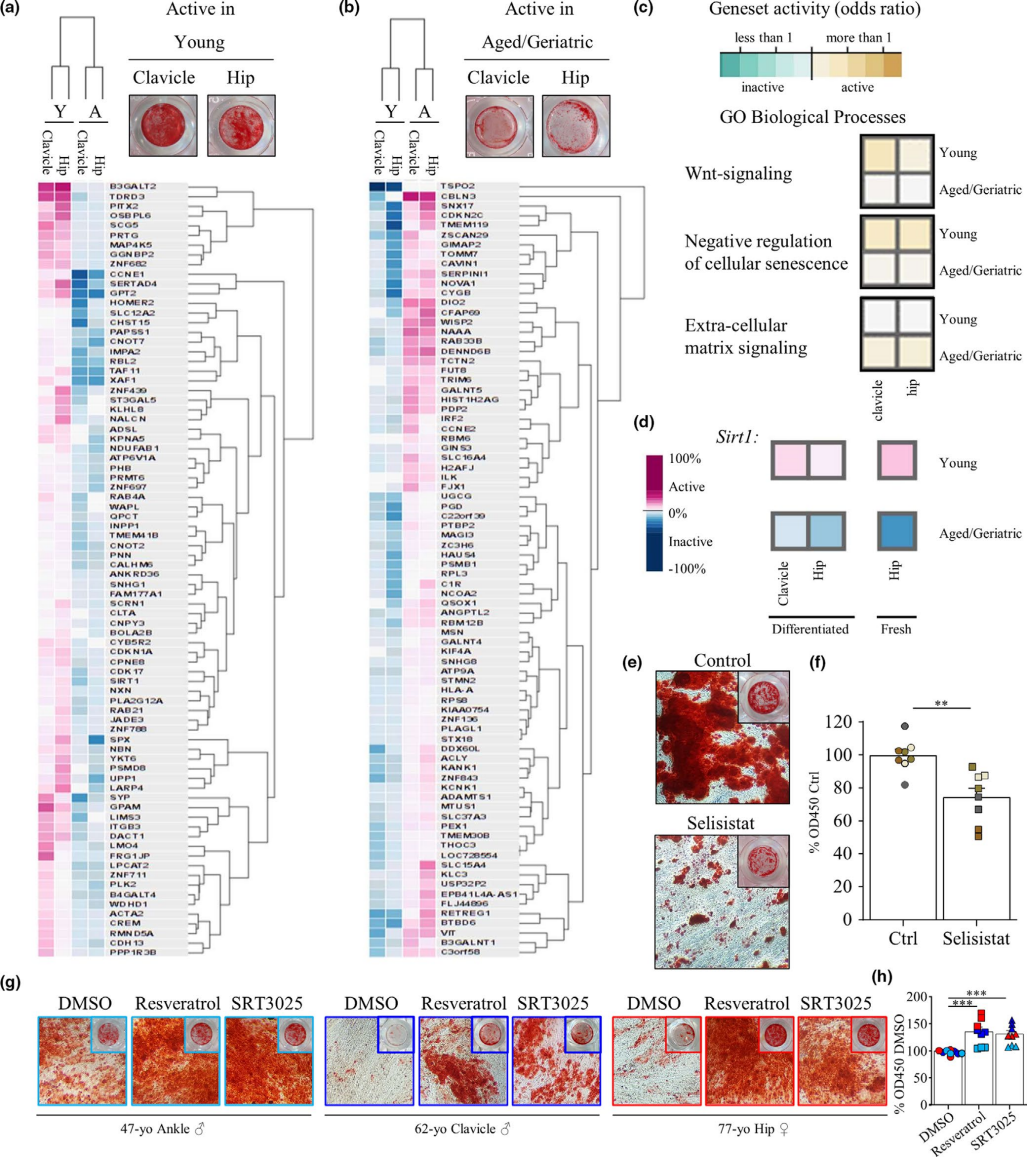
Transcriptomic differences of young and impaired aged hSSCs reveal Sirtuin1 as a potential target for hSSC rejuvenation. (a) Heatmap of microarray data showing the Top 80 genes uniquely active in young (29 and 26 years old) well‐differentiated (top panel: Alizarin Red S) or (b) age‐impaired (49 and 91 years old) ill‐differentiated (top panel: >45 years, Alizarin Red S) hSSCs from clavicle and hip fractures, respectively, as selected by highest dynamic range in dataset by GEXC. Hierarchical clustering of cell types and genes are shown. (c) Geneset activity of GO Biological Processes using GEXC showing either genesets uniquely active in young or aged/geriatric differentiated hSSCs. (d) Sirtuin1 gene expression as assessed by microarray in young hSSCs with strong osteogenic potential and aged hSSCs with impaired osteogenic potential derived from clavicle and hip fracture tissue (left). Sirt1 expression in freshly sorted young or aged hSSCs from hip (right). (e) Representative Alizarin Red S staining images of Sirt1 inhibition with Selisistat (1 µm) during osteogenic differentiation of hSSCs (from clavicle, arm and tibia fracture sites) at day 14. DMSO treated cells served as control. (f) Quantification of osteogenic potential by Alizarin Red S staining (*n* = 8, four different donors). (g) Representative Alizarin Red S staining images of three aged/geriatric patient‐derived hSSCs (two male and one female) with osteogenic differentiation defect (DMSO control) and treated with Sirtuin1 activators trans‐Resveratrol (2 µm) or small molecule SRT3025 (0.2 µm). (h) Quantification of osteogenic potential by Alizarin Red S staining in aged hSSCs from two male (blue) and one female (red) donors (*n* = 9, three different donors). Data shown as mean + standard error of mean (*SEM*). Significance assessed by one‐way ANOVA (***p < .001).

In order to identify targets that could reverse aging of hSSCs, we conducted gene expression analysis on purified hSSCs to determine transcriptomic changes between cells from young and aged/geriatric donors. We isolated RNA of purified hSSCs from young and older individuals from two distinct fracture sites (hip and clavicle) that underwent normal or impaired osteogenic differentiation, respectively (Figure 4a, b). Microarray raw data were submitted to Gene Expression Commons (GEXC) which calculates a percentile value (<0% inactive, >0% active) for each available gene, allowing comparison of human gene expression on a normalized, continuous scale (Seita et al., 2012). Young donor‐derived hSSCs expressed 175 genes that are uniquely elevated relative to aged/geriatric, while 154 genes were specifically elevated in older hSSCs (Table S1). Based on differential gene expression, it is apparent that the hierarchical clustering of young and aged/geriatric samples is very distinct (Figure 4b). Consistent with the osteogenic differentiation defect in geriatric‐derived hSSCs, we observed that skeletogenic Wnt signal pathways and negative regulation of cellular senescence are elevated in young functional hSSCs, whereas older hSSCs were specifically enriched for genes related to extracellular matrix signaling (Figure 4c).

Among the differentially expressed genes that are elevated in young but down in aged/geriatric hSSCs, we identified *Sirtuin1* (*Sirt1*) as a previously reported target implicated in rejuvenation of other tissues and cell types (Stacchiotti, Favero, & Rezzani, 2018) (Figure 4d). Importantly, downregulation of *Sirt1* expression with age was not restricted to in vitro expanded hSSCs but was also apparent in freshly FACS isolated hip fracture‐derived hSSCs. We therefore first asked whether inhibition of Sirt1 by the highly selective inhibitor Selisistat (1 µm) decreased osteogenic differentiation in hSSCs with functional mineralization capacity. Analysis of fracture‐derived hSSCs from four different patients uniformly showed a decline in osteogenic potential as measured by Alizarin Red S staining when Sirt1 was inhibited (Figure 4e,f). Strikingly, supplementing the osteogenic cocktail with a naturally occurring Sirt1‐activator, trans‐Resveratrol (2 µm), or a specific small molecule, SRT3025 (0.2 µm), was sufficient to significantly increase osteogenic differentiation in otherwise functionally impaired hSSCs derived from female and male donors (Figure 4g,h). This positive effect seemed to be more pronounced in hSSCs from older patients who exhibit a more severe state of functional deterioration. Altogether, by profiling transcriptomic differences between functionally distinct young versus older hSSCs, we were able to identify Sirt1 activation as a means to improve the differentiation capacity of impaired hSSCs. Sirt1 agonists could conceivably be an efficacious therapy to prevent or treat impaired fracture healing in geriatric patients.

## 3 DISCUSSION

We have previously described a highly purified long bone hSSC and examined age‐related changes in hSSCs of hip fractures (Chan et al., 2018). Here, we confirmed the presence of hSSCs atfracture sites in all ages investigated and extended that finding to multiple distinct anatomic regions (i.e., upper extremity, tibial plateau, and ankle). These results were gathered from a large number of human samples (*n* = 61) and, to our knowledge, represent the first specific characterization of bona fide SSCs in human fracture tissue. Earlier studies of functionally analogous mouse SSCs show that these cells accumulate in callus tissue, peaking at around 10 days post injury (dpi) before decreasing in number during hard callus remodeling (Chan et al., 2015; Lageneste et al., 2018; Marecic et al., 2015). Consistent with these findings, we also found a correlation between elevated hSSC prevalence and increasing time from injury. Although we did not observe a decrease of hSSCs in fracture calluses during the time frame that we examined, this could be explained by the much slower healing rate in humans as compared to mice and the fact that specimens were obtained from fracture surgeries conducted in the early phases of healing (Einhorn, 2005). Interestingly, not all investigated anatomic regions showed a significant correlation between hSSC number and dpi (e.g., arm). This might reflect region‐specific healing rates, but larger sample sizes will be needed to validate this theory.

Unexpectedly, the frequency of hSSCs at fracture sites was unaltered with age. This is in contrast to a recent finding that reported skeletal stem/progenitor cell frequency declines with age (Josephson et al., 2019). However, that study used a much less stringent marker profile, based solely on a population with negative expression of CD45 and positive expression of CD271, which has not systemically been tested for bona fide stem cell characteristics and, at best, enriches for stem/progenitor cells. In the hematopoietic system, it seems that stem cell numbers are maintained and even increase with age, although there are age‐related functional defects including altered/biased downstream lineage output (Pang et al., 2011). Similarly, we found in hSSCs that osteochondrogenic differentiation capacity was significantly reduced with increasing patient age. This suggests a direct connection between hSSCs and age‐related decline in skeletal health, including slower healing, higher complication rates, and overall poorer outcomes (Demontiero et al., 2012). Importantly, our data are the first to describe a more pronounced aging phenotype in hSSCs isolated from women, which may explain the higher rate of fragility fractures and increased complication during recovery in post‐menopausal women relative to men (Kempen et al., 2003; Seeman, 2001). While changes in innate immunity and acute inflammation have been suggested as contributing factors in aberrant fracture healing (Josephson et al., 2019), it is plausible that these effects are also linked to changes in hSSCs, which in addition to bone and cartilage also generate fibroblastic hematopoietic‐supportive stromal cells. Our results suggest that geriatric hSSCs preferably acquire a skewed differentiation fate, i.e. fibrogenic, which might further impair the healing process. Since hSSCs are still detectable at high numbers in acute fractures of older patients, it is very likely that they might maintain an undifferentiated state and therefore it may be possible to intervene early by identifying factors that can stimulate osteogenic differentiation.

We may have identified one such candidate by analyzing differentially expressed genes in hSSC between young and geriatric patients. As we had anticipated, osteogenic pathways such as Wnt were upregulated in young hSSCs and downregulated in older SSCs (Day, Guo, Garrett‐Beal, & Yang, 2005; Goodnough et al., 2014). Genes involved in suppressing cellular senescence were also repressed in older hSSC. Notably, *Sirt1,* which has been proposed to increase stress resistance and cell death protection expression, was downregulated in geriatric hSSCs. This is mechanistically interesting as Sirt1 functions as a histone deacetylase which implies that functional differences in hSSC may be epigenetically regulated during aging. Moreover, as studies in mammals have shown lifespan extension and delay of aging by preserving Sirt1 expression (Stacchiotti et al., 2018), activation of Sirt1 can be achieved with natural compounds and small molecule drugs and is generally well tolerated which makes it an attractive target. Several recent studies in less well‐characterized cell populations of mice and humans have also shown that activation of Sirt1 can promote a pro‐osteogenic phenotype (Hou et al., 2019; Sun et al., 2018; Tseng et al., 2011; Wang et al., 2019). Therefore, targeted activation of Sirt1 could mitigate bone loss and enhance fracture healing in elderly people while simultaneously alleviating other age‐related conditions. Future studies could reveal the mechanism through which hSSC aging occurs and whether interventions such as Sirtuin1 re‐activation lead to a molecular rejuvenation of hSSC or acts by promoting the activity of downstream hSSC lineage‐committed bone or cartilage progenitors.

Since this study assessed stem cells in fracture healing in humans with the purpose of providing insights into a clinically relevant problem, it was not feasible to test whether hSSCs are necessary or sufficient for fracture healing in vivo. The rate of non‐union is relatively low overall in humans, and therefore, a very large number of patients would be needed to correlate SSC characteristics with clinical healing outcomes in this initial characterization. Assessment of intermediate healing outcomes, such as time to healing on plain radiography, was also not feasible as variable patient follow‐up often confounds this as a routine measure of healing time. Another limitation is that lineage tracing is not feasible in humans, and we previously demonstrated that hSSCs isolated or generated from multiple human tissue sources can be prospectively isolated, maintain clonogenicity, and undergo multi‐lineage skeletal differentiation. Within these limitations, therefore, the presented group of labeled markers presents the most specific panel to isolate bona fide hSSCs to date.

In summary, our results demonstrated that compromised healing in geriatric fractures might underlie age‐related hSSC defects. Contrary to our expectations, injury‐dependent expansion of hSSCs did not show a significant age‐related decline and the significant loss of colony‐forming potential in hSSCs isolated from older patients was sex‐dependent. Instead, we discovered that geriatric hSSCs demonstrate significant osteochondrogenic differentiation defects, which is also reflected in the diminished expression of pro‐osteogenic signaling as well as defects in anti‐aging pathways. We also found that some of the consequences of hSSC aging may be reversible as agonists to the Sirt1 histone deacetylase significantly improved osteogenic differentiation of age‐impaired hSSCs. This is the first investigation into the role of purified and functionally characterized hSSCs, which suggests that age‐related defects in fracture healing could be molecularly diagnosed and treated at the stem cell level in humans.

## 4 EXPERIMENTAL PROCEDURES

### 4.1 Human samples

Sixty‐one specimens of callus tissue from fractured bones (13–94 years old) were obtained during open reduction internal fixation of fractures at Stanford Hospital and collected in accordance with guidelines set by the Institutional Review Board (IRB‐35711). No restrictions were made regarding the race, sex, or age of the specimen’s donor. Only fractures that were undergoing an open approach and direct reduction of fracture fragments were included. Impeding hematoma and fracture callus tissue that disturb satisfactory reduction (alignment) of displaced fragments were removed and saved for research purposes at time of surgery. In each instance, only hematoma/callus which was interposed between the two ends of the fracture was removed in order to reassemble/reduce fragments prior to fixation. Intramedullary sampling and periosteal stripping were excluded from this study. Following excision, all specimens were placed on ice, and hSSCs were isolated within 5 hr upon surgery as described below. Specimen was grouped by their source of origin and included ankle, arm (humerus, ulna, and olecranon), clavicle, hip (femoral head and acetabulum), and tibial plateau.

### 4.2 Isolation of hSSCs

Human callus tissue samples were washed in ice‐cold phosphate‐buffered saline (PBS). If present, clotted blood was gently dissected away before mincing specimens with a razor blade or bone scissors. Minced tissue was immediately placed into 3,000 U/ml type II collagenase (Sigma‐Aldrich, Cat#C6885) digestion buffer supplemented with 100 U/ml DNase I (Worthington, Cat#NC9199796) and incubated at 37°C for 60 min under constant agitation. The supernatant was filtered through a 70 nm nylon mesh and quenched with FACS staining media (2% fetal bovine serum [FBS], 1% Penicillin‐Streptomycin solution [GIBCO, Cat#15140122], 0.1% Pluronics [GIBCO, Cat#24040032], in phosphate‐buffered saline [PBS‐GIBCO, Cat#C14190500BT]). Filtered cells were then centrifuged at 200 g at 4°C for 5 min. The supernatant was discarded, and the pellet resuspended in ACK lysis buffer (GIBCO, Cat#A10492‐01) to remove red blood cells. The reaction was stopped by filling the tube with staining media, and centrifugation was repeated. The supernatant was discarded, followed by resuspension in a small amount of staining media. The resuspended pellet was stained with fluorochrome‐conjugated antibodies against CD45 (BioLegend, Cat#304029‐BL), CD235a (BioLegend, Cat#306612‐BL), CD31 (Thermo Fisher Scientific, Cat#13‐0319‐82), CD202b (Tie‐2) (BioLegend, Cat#334204), CD146 (BioLegend, Cat#342010), PDPN (Thermo Fisher Scientific, Cat#17‐9381‐42), CD90 (THY1; BioLegend, Cat#328110), CD164 (BioLegend, Cat#324808), and CD73 (BioLegend, Cat#344016). Flow cytometry was performed on a FACS Aria II (BD Biosciences). Gating schemes were established with fluorescence‐minus‐one (FMO: staining with all fluorophores except one) controls, and negative propidium iodide (Sigma‐Aldrich, Cat#P4170) staining (1 mg/ml) was used as a measure for cell viability.

### 4.3 In vitro cell culture

Human SSCs were FACS sorted and seeded on 10 cm plastic dishes containing MEM‐alpha medium with 10% human platelet‐derived lysate (Stem Cell Technologies, Cat#06960) and 1% Penicillin–Streptomycin solution (Pen‐Strep; Thermo Fisher Scientific, Cat#15140‐122). Cells were maintained in an incubator set to 37°C with 5% CO_2_. For colony‐forming assays (CFU‐F), hSSCs were seeded at clonal density and cultured for 10–14 days, fixed in 4% formalin and stained with Crystal Violet (Sigma‐Aldrich, Cat#C0775) for counting. Freshly sorted and expanded cells at sub‐confluency were lifted and re‐seeded for differentiation assays. Upon 100% confluency, osteogenic factors were added to culture medium containing MEM‐alpha medium (Thermo Fisher Scientific, Cat#12561‐056), 10% FBS, 1% Pen‐Strep, 100 nm dexamethasone (MP Biomedicals, Cat#194561), 10 mm sodium b‐glycerophosphate (Sigma‐Aldrich, Cat#G9891), and 2.5 mm ascorbic acid 2‐phosphate (Sigma‐Aldrich, Cat#A8960). Media was changed every other day, and cells were fixed with 4% formalin at day 14 of differentiation culture for staining with Alizarin Red S (Sigma‐Aldrich, Cat#A5533). For experiments involving Sirt1‐inhibition and activation DMSO (control), Selisistat (1 µm; Selleckchem, Cat#S1541), trans‐Resveratrol (2 µm; Cayman Chemical, Cat#70675), or SRT3025 (0.2 µm; Selleckchem, Cat# S8481) were added to the differentiation cocktail. Chondrogenesis assays were conducted by micromass assay. Briefly, cells were resuspended at a cell density of 1.6 × 10^7^ cells/ml. A 5 µl droplet of the cell suspension was seeded under high humidity conditions in a 24‐well plate for 2 hr. After 2 hr, warmed chondrogenic differentiation media was added to the culture vessel. The growing micromass was fed with fresh chondrogenesis media (DMEMhigh [Thermo Fisher Scientific, Cat# 10569010] with 10% FBS, 100 nm dexamethasone, 1 µm ascorbic acid 2‐phosphate, and 10 ng/ml TGFβ1 [Peprotech, Cat# 100‐21C]) every other day in a 37°C incubator with 5% CO_2_. At day 10 the micromass was fixed, sectioned, and stained with Alcian blue (Sigma‐Aldrich, Cat#A5268).

### 4.4 Transcriptional expression profiling

Microarray analysis was performed on highly purified, double‐sorted hSSC populations either directly sorted into TRIzol LS (Invitrogen, Cat#10296028) or expanded and differentiated into the osteogenic fate for 14 days before collection in TRizol. RNA was isolated with RNeasy Micro Kit (QIAGEN, Cat#74004) as per manufacturer’s instructions. RNA was twice amplified with an Arcturus RiboAmp PLUS Kit (Applied Biosystems, Cat#KIT0521). Amplified cRNA was streptavidin‐labeled, fragmented, and hybridized to Affymetrix arrays HG‐U133+ (for human genome; Applied Biosystems, Cat#901569). Arrays were scanned with a Gene Chip Scanner 3000 (Affymetrix) running GCOS 1.1.1 software (Affymetrix). Raw microarray data were submitted to GEXC (https://gexc.riken.jp/models/2341). On this platform, data are normalized by computing against the Common Reference, which is comprised of more than 25,229 human array experiments deposited to the National Institutes of Health Gene Expression Omnibus (NIH GEO) database. GEXC assigns a threshold value to each probeset using the StepMiner algorithm and calculates a percentile value between −100% and +100% for each available gene, allowing comparison of human gene expression on a normalized, continuous scale. A list of hSSC gene transcripts specific to young (<45 years old) or aged (>45 years old) donor specimen was generated by using the pattern search feature of the GEXC database. The platform also allows Geneset activity search for GO Biological Processes. The algorithm determines odds ratios of more than one to determine whether a specific Geneset is active or inactive.

### 4.5 Statistical analysis

Statistical analyses were performed on Prism 8.0.2. (GraphPad Software). Linear relations of groups were determined by two‐tailed Pearson correlation coefficient. Bar graphs are shown as mean + *SEM*, and statistical significance between groups was determined by one‐way ANOVA with Fisher's LSD post hoc test for three groups and Tukey's post hoc test for more than three groups.

## CONFLICT OF INTEREST

The authors declare no competing interests.

## AUTHOR CONTRIBUTIONS

T.H.A, L.H.G., and C.K.F.C. conceived the study, analyzed results, and wrote the manuscript. T.H.A. and L‐H.G. performed most experiments. H.M.S., M.Y.H., L.S.K., E.K., O.M., J.S., J.A.B., and M.J.G. contributed to experiments. C.K.F.C. supervised the project.

## Supporting information

Figure S1

Figure S2

Table S1

## Data Availability

The microarray data reported in this paper are publicly accessible at https://gexc.riken.jp/models/2341.
