# Supplementary figures and images for: Geriatric fragility fractures are associated with a human skeletal stem cell defect
Source: Aging Cell. 2020 Jun 14;19(7):e13164. doi: 10.1111/acel.13164 (PMC7370785; doi:10.1111/acel.13164)

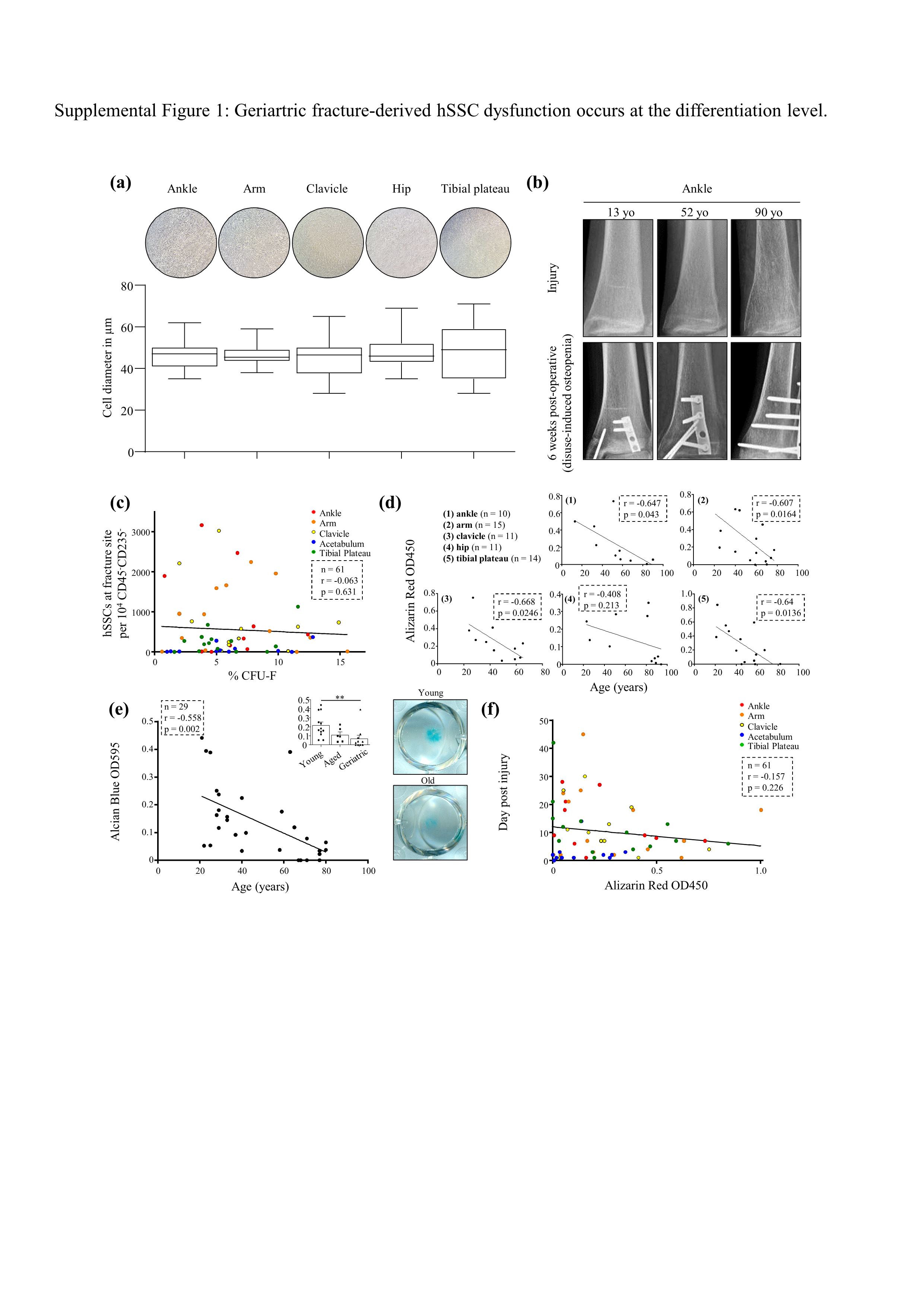

Supplement: Supplementary file 1 — Figure S1 [file ACEL-19-e13164-s001.tif]

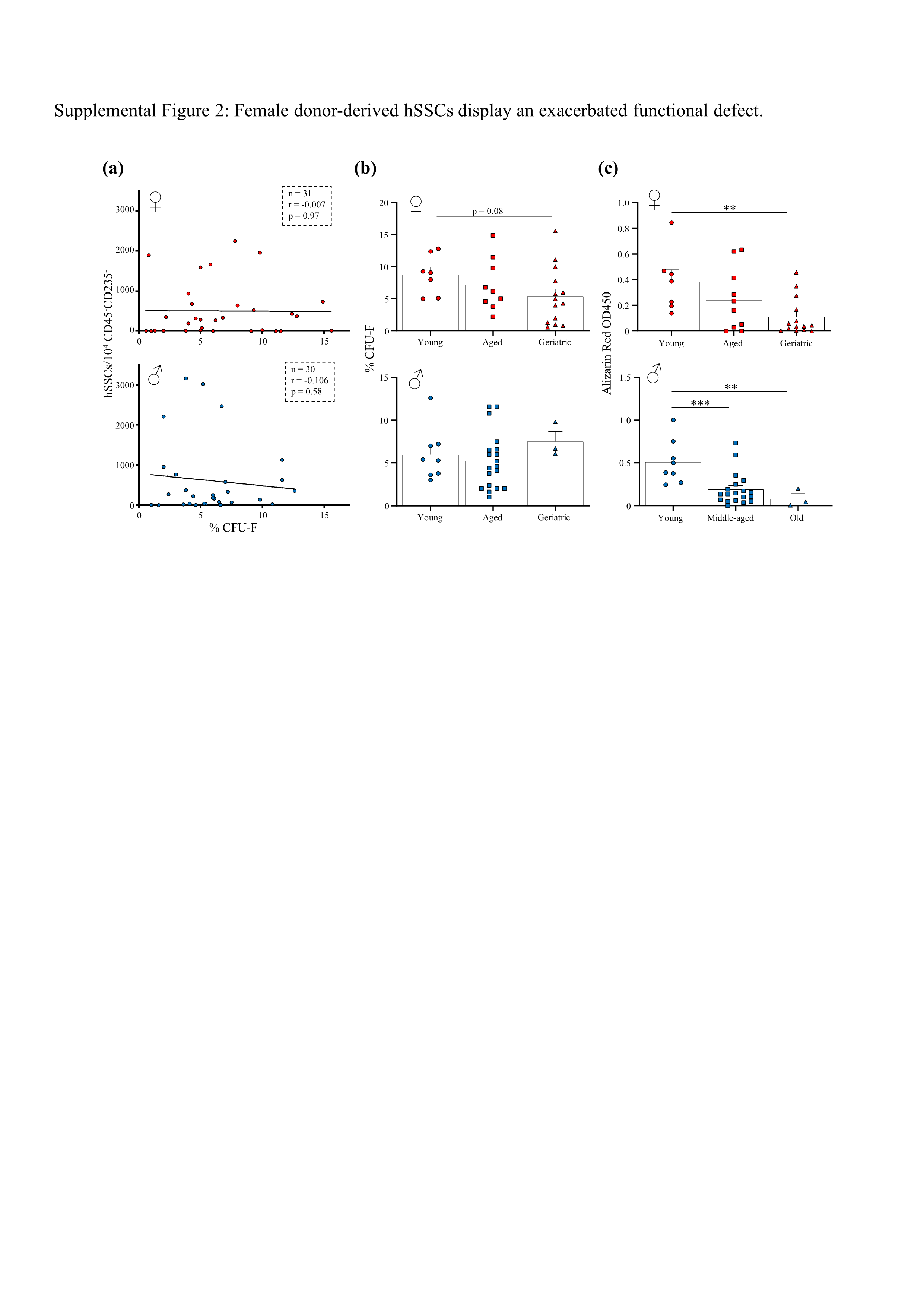

Supplement: Supplementary file 2 — Figure S2 [file ACEL-19-e13164-s002.tif]
